# Investigating the role of auditory and visual sensory inputs for inducing relaxation during virtual reality stimulation

**DOI:** 10.1038/s41598-022-21575-9

**Published:** 2022-10-12

**Authors:** Aileen C. Naef, Marie-Madlen Jeitziner, Samuel E. J. Knobel, Matthias Thomas Exl, René M. Müri, Stephan M. Jakob, Tobias Nef, Stephan M. Gerber

**Affiliations:** 1 Gerontechnology and Rehabilitation Group, ARTORG Centre for Biomedical Engineering Research, University of Bern, Bern, Switzerland; 2 Department of Intensive Care Medicine, Inselspital, Bern University Hospital, University of Bern, Bern, Switzerland; 3 Department of Public Health, Institute of Nursing Science, Faculty of Medicine, University of Basel, Basel, Switzerland; 4 Department of Neurology, Inselspital, Bern University Hospital, University of Bern, Bern, Switzerland

**Keywords:** Biomedical engineering, Stress and resilience

## Abstract

Stress is a part of everyday life which can be counteracted by evoking the relaxation response via nature scenes presented using immersive virtual reality (VR). The aim of this study was to determine which sensory aspect of immersive VR intervention is responsible for the greatest relaxation response. We compared four conditions: auditory and visual combined (audiovisual), auditory only, visual only, and no artificial sensory input. Physiological changes in heart rate, respiration rate, and blood pressure were recorded, while participants reported their preferred condition and awareness of people, noise, and light in the real-world. Over the duration of the stimulation, participants had the lowest heart rate during the audiovisual and visual only conditions. They had the steadiest decrease in respiration rate and the lowest blood pressure during the audiovisual condition, compared to the other conditions, indicating the greatest relaxation. Moreover, ratings of awareness indicated that participants reported being less aware of their surroundings (i.e., people, noise, light, real environment) during the audiovisual condition versus the other conditions (p < 0.001), with a preference for audiovisual inputs. Overall, the use of audiovisual VR stimulation is more effective at inducing a relaxation response compared to no artificial sensory inputs, or the independent inputs.

## Introduction

A recent survey has found that roughly three-quarters of American adults reported experiencing physical or psychological stress^1^, with older individuals found to be more vulnerable to stressors^2^. Stress represents the body’s reaction to any natural stimulus that evokes a physical or psychological response in the body away from homeostasis^3,4^. Although not innately harmful, prolonged exposure to stress can result in a variety of negative health outcomes (e.g., anxiety, cardiovascular diseases, gastrointestinal inflammation) and, in turn, can result in a high economic burden^4–7^. The body can counteract the stress response by evoking the relaxation response, a process which results in a number of physiological changes^8–10^. Specifically, the body’s metabolism will lower, while decreasing heart rate, respiration rate, and blood pressure, which is all opposite the body’s response to stress^9,11^. Such a relaxation response can be elicited via exposure to nature, as supported by the literature^8–10^.

The notion that nature can elicit a relaxation response and increase overall well-being has been well studied^12–17^. Whereas initial studies investigated people who were physically exposed to natural environments, more recent studies have been investigating if the same beneficial effects of nature can be achieved artificially^18–22^. This work has found that nature presented via virtual methods (i.e. virtual environments and immersive VR) has a relaxing effect^18,19,23–26^, and that nature presented via immersive VR results in a greater relaxation response than nature presented via a standard television^27,28^.

During audiovisual immersive VR the goal is to effectively substitute the sensory perception of the real world with the sensory perception of the virtual world^29^. In this way, consciousness of the real world is transferred to a consciousness of the virtual world^29^. However, even with current state-of-the-art immersive VR technology, it is only possible to stimulate a subset of one’s senses, allowing any senses which are not virtually stimulated to be stimulated by the real world^30,31^. This occurrence is referred to as a misalignment^30^. The better the alignment between the real and virtual world, via technology that allows for effective sensory substitution, the higher the level of immersion created^29,32^. In turn, the user will experience a greater sense that what they are experiencing in the virtual world is real, influencing their sense of presence within the virtual environment^30,31,33^. This concept is in line with the findings by Annerstedt et al.^19^ who found that although nature sounds are often reported as being relaxing, more significant effects were found during stress recovery when auditory stimuli was presented together with visual input. However, their work failed to investigate the effects of stress recovery when presented with auditory stimuli alone. Moreover, the work of Annerstedt et al.^19^ presented nature stimuli within a virtual space projected onto the surrounding environment, without the use of a head-mounted display, after exposure to a stressor. Such a setup is not only unfeasible for a large portion of the population who may benefit from relaxing VR nature scenes, such as elderly individuals or hospital patients, but it also fails to account for the fact that one’s environment may be inherently stressful. In such environments, the use of a head-mounted display and noise-cancelling headphones are believed to be important to inhibit individuals from being visually and auditorily aware of their real-world surroundings furthering their sense of “being there” in the virtual environment, thus improving relaxation^34^. This is of particular relevance when considering environments where individuals may experience sensory overload or sensory deprivation^35^. Sensory overload can occur when there is excessive and continuous overstimulation above normal levels of one or more senses^35^. Alternately, sensory deprivation occurs when individuals are exposed to monotonous or minimal stimuli^35,36^. In both environments, immersive VR technology, presented via a head-mounted display and noise-cancelling headphones, could prove to be a promising tool to counteract sensory overload and deprivation, by creating a virtual sense of presence away from their real physical environment.

However, as explained by Slater^32^, simply creating a sense of presence in the virtual environment says nothing about the interest or emotional response elicited by the environment. Rather, this aspect of an environment is dictated by the content shown, which can vary based on the goal of the stimulation. For example, previous work has looked at using virtual reality as a distraction tool to target different aspects, such as boredom or pain, in the hospital setting^37–40^. Such setups would require a specific type of content, perhaps a game or a video with a lot of changing content or aspects to virtually explore, keeping the user’s attention firmly on the virtual world. Alternately, to achieve relaxation as is the goal of the current study, calm nature content was selected to promote relaxation. This specifically addresses the question of content, and should be considered independently of the level of presence achieved as described by Slater^32^.

Unfortunately, to our knowledge, there is no research that addresses which component of immersive VR, visual, auditory, or the combination of the two, is responsible for the relaxing effect described in the literature^8–10^. Therefore, the aim of this study was to fill this gap, investigating which component of immersive VR leads to the greatest relaxation effect. In this study, four different conditions, representing audiovisual inputs, auditory only or visual only inputs, and no artificial sensory inputs, were presented to healthy participants while recording objective stress levels, and subjective preference and awareness. We hypothesize that the greatest relaxation defined as a decrease in heart rate, respiration rate, and blood pressure, will be elicited when the content is presented simultaneously (i.e., audiovisual condition) via the head-mounted display with noise-cancelling headphones. Moreover, it is predicted that alignment between the sensory components enabled via the simultaneous audiovisual input will allow users to become immersed in the virtual world, enabling them to enjoy their immersive VR experience while decreasing their awareness of the real world.

## Methods

### Participants

A total of 42 (25 female, 17 male) healthy individuals participated in the study and ranged in age between 24 and 83 years (mean 60.2, SD 15.3). Participants were recruited via email and word of mouth. The study protocol and information were explained to each participant verbally, and a written informed consent was obtained prior to participation on the first day. The main exclusion criteria were age less than 18 years, self-reported visual and auditory impairments (i.e., not normal, and not corrected-to-normal), and non-German speaking. The study was approved by the Ethics Committee of the Canton of Bern, Switzerland (KEK no. 2017-02195), and carried out in accordance with the current version of the Declaration of Helsinki.

### Study design and procedure

This interventional crossover study was conducted in a research room, located within the University Hospital of Bern. The room was equipped with all standard hospital equipment, monitors, and auditory alarms. The study involved four individual sessions, with participants asked to take part on four separate days within a two-week period. Over the course of these four days every participant received each of the four test conditions once (Table 1). The order in which the conditions were presented was counterbalanced and randomly assigned. Every possible sequence of conditions was tested at least once, with no sequence of conditions being tested more than twice.

**Table 1:** Descriptions of the four different test conditions.

Sensory inputs (*condition)*	Devices used	Condition description
Audiovisual *(AV)*	Head-Mounted Display & Noise-Cancelling Headphones	Participants received both the 360° video and corresponding sound through the head-mounted display and noise-cancelling headphones
Auditory only *(A only)*	Noise-Cancelling Headphones Only	Participants received only the audio through the noise-cancelling headphones without wearing the head-mounted display
Visual only *(V only)*	Head-Mounted Display Only	Participants received only the 360° video visually through the head-mounted display without wearing the noise-cancelling headphones
Control	No Head-Mounted Display & No Noise-Cancelling Headphones	Participants did not receive any video or sound and did not wear the head-mounted display nor the noise-cancelling headphones. Participants were not blindfolded and did not wear earplugs.

Upon arrival, participants were briefed on the relevant information for their participation specifically in relation to the given intervention condition for that day. Subsequently, participants were prepared for the recording of their vital signs. A vital sign monitoring system (Carescape Monitor B650, GE Healthcare, Little Chalfont, UK) was used in combination with a five-lead electrocardiogram and a cuff-based blood pressure monitor. Heart rate and respiration rate were recorded as the mean every 5 s, while the non-invasive mean arterial blood pressure was recorded every 2 min. The entire preparation took approximately 15 min and was done while the participant was first calmly seated in a chair, and then laying on a bed. Once recording of the vital signs began, the intervention was started and lasted 30 min, with no interaction with the study team during this time. Depending on the condition, this involved 30 min of video, audio, both combined, or nothing at all (Table 1). Each participant was randomly assigned auditory and visual input material, consisting of a single 360° video and corresponding sound. The same material was used across all test sessions, with only the corresponding video or sound provided during the independent auditory and visual conditions (Table 1). Environmental consistency was achieved across participants and conditions by controlling multiple factors. During the 30-min intervention there were always two members of the study team present in the room, one person entered and left the room, three high-level alarms occurred (one per 10-min segment), multiple mid-level alarms occurred, and one flashing red light was turned on for 2 min. The same default sound pressure level was used across all participants and conditions for the high (73 dBA) and mid (69 dBA) level alarms. Participants were instructed to do whatever felt natural to them during this time, and not to force any particular behavior (e.g., keeping their eyes open, trying to identify the sounds, remaining still). This approach was selected to avoid causing a stress response as a result of participants feeling as though they had to act a certain way.

After the 30-min intervention period had elapsed, participants were asked to complete a general questionnaire asking about their awareness of the real world (i.e., people, noise, light, real environment) during the intervention period (Table 2). Questions asked were developed specifically for this study and were adapted from questions from previously validated questionnaires^41,42^. Validated questions regarding presence and immersion were also asked (Supplementary Table S1)^42–44^. On the final day the participants were additionally asked to report which of the four conditions was their preferred intervention.

**Table 2:** Description of Subjective Participant Awareness and Intervention Preference Questions (translated from German). Q1 was asked after each condition except when no artificial sensory inputs were provided. Q2-5 were asked after each of the interventions, and Q6 was asked only after the final intervention was completed.

		Not at all	Slightly	Somewhat	Moderately	Extremely
Q1	During my experience I felt unwell (e.g., nausea, dizziness, …)	**☐**	**☐**	**☐**	**☐**	**☐**
Q2	I was aware of my real surroundings	**☐**	**☐**	**☐**	**☐**	**☐**
	I was aware of the following things:					
Q3	Light *(warning signals, room lighting, …)*	**☐**	**☐**	**☐**	**☐**	**☐**
Q4	Noise *(sounds of equipment, alarms, opening of packages, …)*	**☐**	**☐**	**☐**	**☐**	**☐**
Q5	Staff *(coming and going, conversations, …)*	**☐**	**☐**	**☐**	**☐**	**☐**

		Audio and visual	Auditory only	Visual only	Control	
Q6	Which condition did you prefer the most?	☐	☐	☐	☐	

### Stimulation material and apparatuses

**Figure 1:**
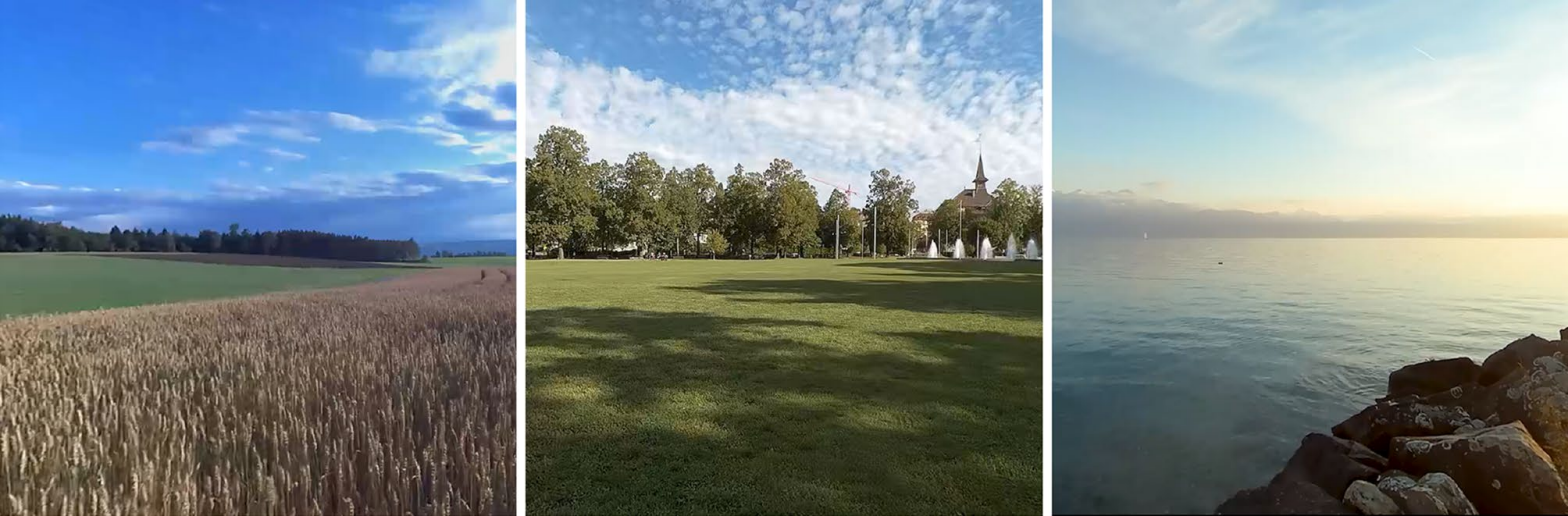
Still photo from three of the videos provided to the participants via the head-mounted display.

A total of 42 unique videos were shown to the participants. All videos presented were 360° videos, showing a single continuous video. The videos were recorded using a commercially available 360-degree camera (Insta360 Pro 2, Insta306, Shenzhen, China) and standard video editing software (Adobe Premiere Pro) to create the final videos. All videos depict locations within Switzerland and focus on calm nature environments (e.g., an open field, a park, a lake with waves). Sounds overlaying the videos are recordings of nature sounds spliced together to make a soundscape fitting the scene presented in the video. The goal of the sounds was to match the scene in the video, while remaining calm (e.g., waves lapping at the shore, birds chirping, wind rustling leaves) (Fig. 1). No distinguishable talking or urban sounds can be heard in the videos. Sound files overlaying the videos had a sampling frequency of 48 kHz. No advanced processing of the sound files to include stereo spatialization or varied auditory distance or elevation was done.

Videos were shown using the Oculus Quest (Oculus VR LLC, Irvine, USA), a high resolution, stand-alone, head mounted display. The headset has a resolution of 1440 × 1600 pixels per eye and a refresh rate of 72 Hz, with the Oculus Quest able to play videos up to 5.7 K. Therefore, the minimum resolution of the videos was set to be at least 5 K (5120 × 2560), with a sampling rate of 24 Hz to ensure no flickering. Videos were played using the integrated Oculus Video app, allowing for an immersive 360° experience. Sounds were played over a wired connection using binaural Sony WH-1000XM3 noise-cancelling headphones. Participants were allowed to adjust the volume of the auditory stimulation to be at a comfortable level for them. To limit cyber sickness, all videos were filmed from a stationary position. This was verified by asking participants via questionnaire if they felt unwell (e.g., nausea, dizziness) due to the VR stimulation. This question was asked after each condition, except when no artificial sensory input was provided (Table 2).

### Data pre-processing

To reduce random noise in the data, a 20 s moving average filter was applied to all the physiological data. Next, as not all participants have the same resting heart rate, respiration rate, and mean arterial blood pressure, the data was centered. To center the data while maintaining the largest dataset possible, the mean of the first three datapoints, representing the first 15 s of the recording, were subtracted from the dataset for each participant. For the mean arterial pressure, which was recorded every 2 min, only the first datapoint was subtracted from the dataset for each participant.

### Statistical analysis

To determine whether or not a relaxing effect was seen in the physiological data over time in the different conditions, the well-established Generalized Additive Mixed Model (GAMM) method was used^45,46^. Within the GAMM model, a thin plate regression spline (ts), with a modification to the smoothing penalty, was specified as the smooth term^46,47^. The physiological parameter of interest (i.e., heart rate, respiration rate, blood pressure) was considered the dependent variable, while time was added as a smoothed fixed effect. The model was adjusted for age and sex, and the participant id was specified as the random intercept. Randomization was not added to the model to avoid adding a spurious association to the model.

As part of the GAMM method the interaction between the different fixed effects and adjustment parameters was calculated. Specifically, the effect of time and the effect of the interaction between time and condition were also investigated. These secondary effects were examined based on the results of a maximum likelihood ratio test which showed strong evidence that there are differences in the smooth functions of the model. A significant interaction term between time and condition indicates that the parameter of interest did not remain stable in its change over the duration of the experiment.

Based on the questionnaires, to determine if there was a subjective difference in the participants’ general awareness of people, noise, light, and the real environment, during the four different conditions, a non-parametric Friedman test was used. Post-hoc analyses were conducted using a Bonferroni corrected, pairwise Wilcoxon signed rank test between groups. Due to small sample sizes when separated for age and sex, no statistical analyses were conducted taking these factors into account.

All analyses were conducted using MATLAB 2019a (MathWorks, Natick, USA) and R for statistics (The R Foundation, Vienna, Austria).

## Results

### Objective measures

**Figure 2:**
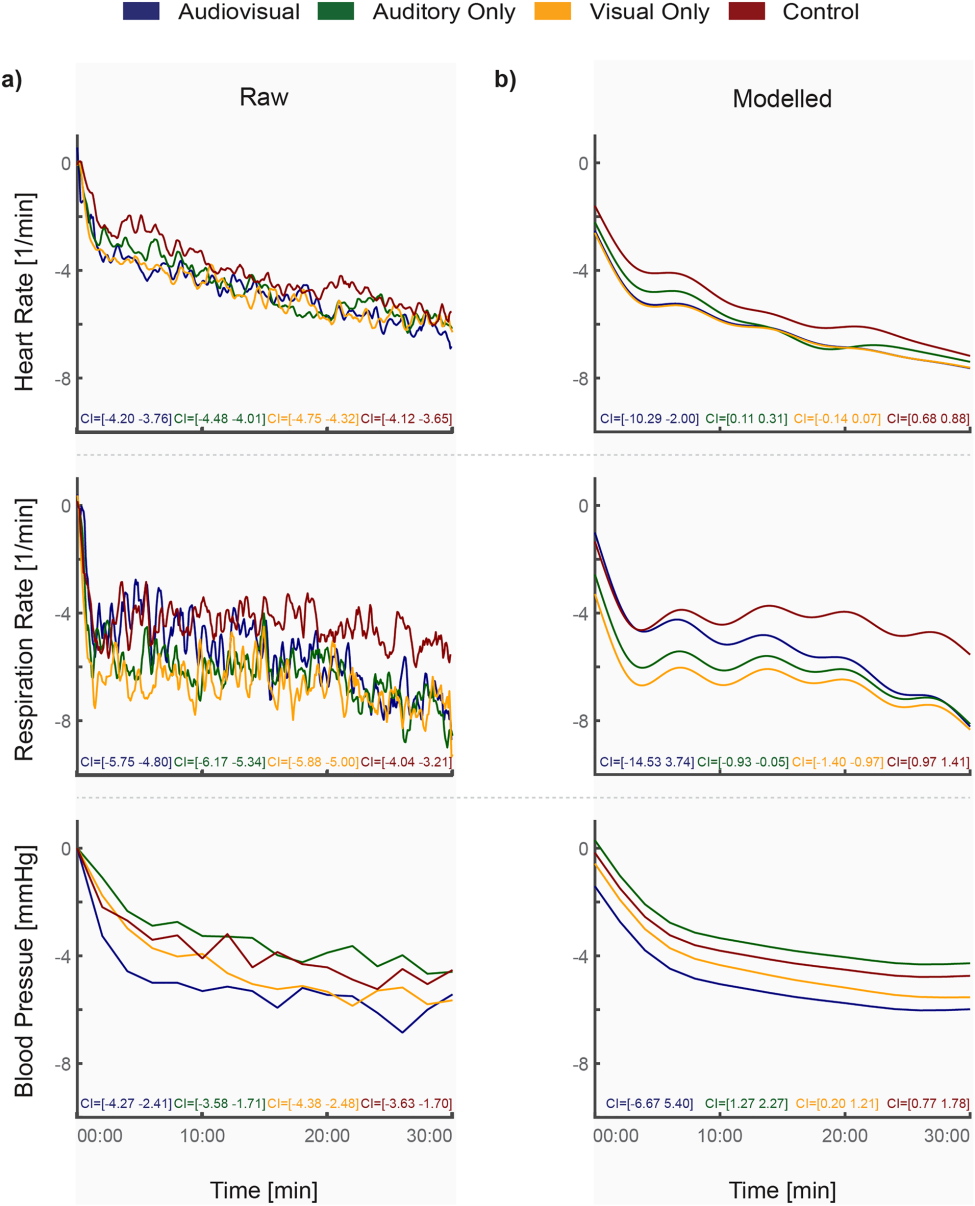
Physiological changes in heart rate (top), respiration rate (middle), and mean arterial pressure (bottom) over the 30-min intervention period. (Left) Raw data (n = 42) which has been centered to zero. 95% confidence intervals (CI) are shown at the bottom of each plot. (Right) Modelled data generated using the Generalized Additive Mixed Model and the raw data. 95% confidence intervals (CI) are shown at the bottom of each plot.

The results of the GAMM (Heart Rate, R^2^ (adj) = 0.054; Respiration Rate, R^2^ (adj) = 0.014; Blood Pressure, R^2^ (adj) = 0.080) have shown significant differences between a number of conditions when looking at the three physiological parameters (Table 3, Fig. 2). Compared to the audiovisual condition, heart rate shows a significant difference between the auditory only and control conditions (A Only, t = 4.051, P < 0.001; Control, t = 14.960, P < 0.001). Compared to the audiovisual condition, respiration rate shows a significant difference in the auditory only, visual only, and control conditions (A Only, t = − 6.488, P < 0.001; V Only, t = − 10.707, P < 0.001; Control, t = 0.700, P < 0.001). Blood pressure shows a significant difference in the auditory only, visual only, and control conditions (A Only, t = 6.883, P < 0.001; V Only, t = 2.734, P = 0.006; Control, t = 4.953, P < 0.001), compared to the audiovisual condition.

**Table 3:** Generalized additive mixed model (GAMM) for the heart rate, respiration rate, and blood pressure. For each model, the coefficient representing the intercept of the data and its significance is shown. The sampling frequency is 12 samples/min for heart rate (t(59,951)) and respiration rate (t(59,951)), and 0.5 samples/min for blood pressure (t(2502)). Intercept data has no practical meaning as the model could not be set to zero^48,49^. Abbreviations: standard error (SE), confidence interval (CI), test statistic with sample size used for the predictions in the model (t( )), proportion of the t distribution at that df which is greater than the absolute value of your t statistic (PR( >|t|)). Significant p-values are in bold.

	Coefficient	SE	95% CI	t	Pr( >|t|)
**Heart rate**					
Audiovisual (intercept)	− 6.142	2.115	[− 10.29, − 2.00]	− 2.904	**0.004**
Auditory only	0.210	0.052	[0.11, 0.31]	4.051	**< 0.001**
Visual only	− 0.034	0.052	[− 0.14, 0.07]	− 0.648	0.517
Control	0.780	0.052	[0.68, 0.88]	14.960	**< 0.001**
Age	0.013	0.026	[− 0.04, 0.06]	0.509	0.610
Sex	0.392	0.805	[− 1.18, 1.97]	0.487	0.626
**Respiration rate**					
Audiovisual (intercept)	− 5.391	4.661	[− 14.53, 3.74]	− 1.157	0.247
Auditory only	− 0.717	0.111	[− 0.93, − 0.05]	− 6.488	**< 0.001**
Visual only	− 1.184	0.111	[− 1.40, − 0.97]	− 10.707	**< 0.001**
Control	1.191	0.111	[0.97, 1.41]	0.700	**< 0.001**
Age	− 0.020	0.057	[− 0.13, 0.09]	− 0.345	0.730
Sex	0.734	1.773	[− 2.74, 4.21]	0.414	0.679
**Blood pressure**					
Audiovisual (intercept)	− 0.634	3.076	[− 6.67, 5.40]	− 0.206	0.837
Auditory only	1.770	0.257	[1.27, 2.27]	6.883	**< 0.001**
Visual only	0.703	0.257	[0.20, 1.21]	2.734	**0.006**
Control	1.272	0.257	[0.77, 1.78]	4.953	**< 0.001**
Age	− 0.030	0.038	[− 0.10, 0.04]	− 0.786	0.432
Sex	− 1.589	1.169	[− 3.88, 0.70]	− 1.360	0.174

Looking at an example, if we take the auditory only condition of the heart rate data, we see that its coefficient is 0.210, with a 95% CI of [0.11, 0.31]. This means that the predicted mean value of the auditory only condition is on average 0.210 beats/min higher than the audiovisual condition (intercept). The CI tells us that we can be 95% sure that this predicted mean value falls within the range of 0.11–0.31 beats/min. Finally, the significance of < 0.001 tells us that this predicted value for the auditory only condition is significantly different than that of the audiovisual condition. The fact that that the auditory only coefficient is positive does not indicate that the heart rate is increasing, but rather that the decrease is less steep than in the reference condition (audiovisual). Similarly, if the coefficient is negative (e.g., Respiration rate, auditory only), it means that the trend is slightly steeper compared to the reference condition (audiovisual).

A maximum likelihood comparison, using a linear mixed effects model, showed strong evidence of an interaction between the intervention condition and the duration of the intervention when analyzing the heart rate (P = 0.0313) and respiration rate (P < 0.001) (see Supplementary Table S2). Heart rate showed an interaction in the auditory only and control conditions (A Only, F = 2.86, P < 0.001; Control, F = 3.49, P < 0.001) (see Supplementary Fig. S1). Respiration rate showed an interaction in the audiovisual and control conditions (AV, F = 4.11, P < 0.001; Control, F = 0.68, P = 0.0061) (see Supplementary Fig. S2). No significant interaction was found in the mean arterial pressure (see Supplementary Fig. S3).

### Subjective measures

**Figure 3:**
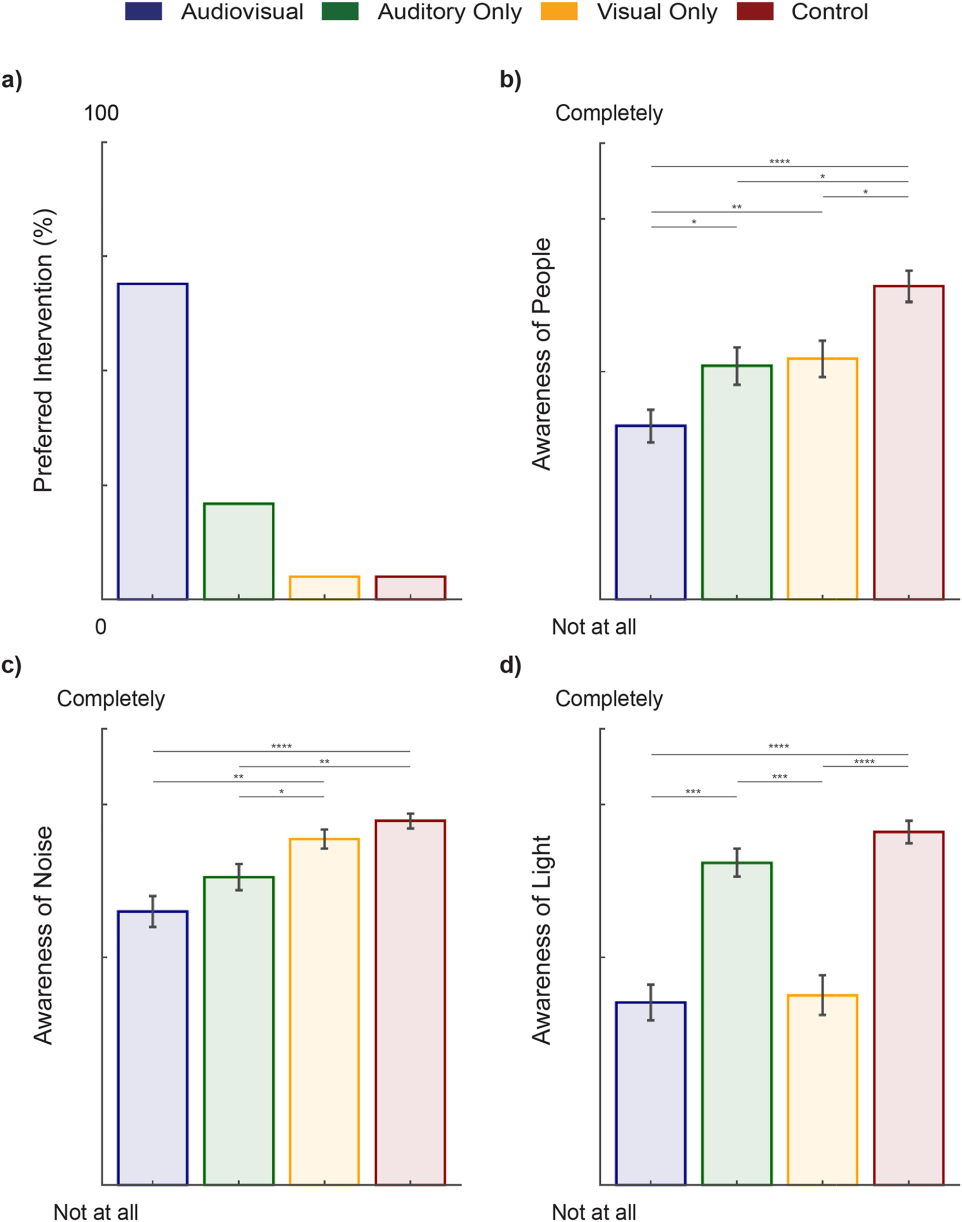
Participant (n = 42) responses to subjective questionnaires. (**a**) Shows the percent of participants who preferred the different conditions. (**b**) Subjective rating of how aware the participants were of people in the room during the different conditions. (**c**) Subjective rating of how aware the participants were of the noises in the room during the different conditions. (**d**) Subjective rating of how aware the participants were of the light in the room during the different conditions. Vertical bars represent the standard error and horizontal bars represent significant differences between the conditions, the alpha level was set to 0.05.

In the subjective questionnaire, the participants reported a clear preference for the audiovisual condition (Fig. 3a). Furthermore, there was a statistically significant difference in general awareness of the real world, as well as awareness of people (χ^2^(3) = 40.1, P < 0.001), noise (χ^2^(3) = 37.3, P < 0.001), light (χ^2^(3) = 60.6, P < 0.001), and the real environment (χ^2^(3) = 22.1, P < 0.001) in the study room, depending on which condition the participant was receiving (Table 4, Fig. 3b–d).

**Table 4:** The difference in subjective awareness between the different intervention conditions. Due to non-normality and use of an ordinal scale for the dependent variable, a non-parametric Friedman test was used. It was followed up by a pairwise Wilcoxon signed rank test, with a Bonferroni correction, to identify which conditions are different. Questions asked are presented on the left (Table 2) and were rated using a Likert scale from 1 (“Not at all”) to 5 (“Completely”). Abbreviations: Friedman test statistic (Statistic), p-value (p), Bonferroni adjusted p-value (p-adjusted). Significant p-values are in bold.

Question	Group 1 (*n* = 42)	Group 2 (*n* = 42)	Test statistic	p	p adjusted
**I was aware of people (coming and going, conversations, …)**	Friedman test *(df* = *3)*		40.1	< 0.001	–
	Auditory only	Audiovisual	245	0.006	**0.037**
	Auditory only	Control	69	0.002	**0.013**
	Auditory only	Visual	187	0.719	1
	Audiovisual	Control	5	< 0.001	**< 0.001**
	Audiovisual	Visual only	40.5	< 0.001	**0.003**
	Control	Visual only	285	< 0.001	**< 0.001**
**I was aware of noise(s) (sounds of equipment, alarms, opening packages, …)**	Friedman test *(df* = *3)*		37.3	< 0.001	–
	Auditory only	Audiovisual	198	0.063	0.376
	Auditory only	Control	28	< 0.001	**0.004**
	Auditory only	Visual	40	0.004	**0.022**
	Audiovisual	Control	0	< 0.001	**< 0.001**
	Audiovisual	Visual only	31.5	< 0.001	**0.001**
	Control	Visual only	73.5	0.188	1
**I was aware of light(s)** **(warning signals, room lighting, …)**	Friedman test *(df* = *3)*		60.6	< 0.001	–
	Auditory only	Audiovisual	549	< 0.001	**< 0.001**
	Auditory only	Control	25	0.025	0.149
	Auditory only	Visual	562	< 0.001	**< 0.001**
	Audiovisual	Control	0	< 0.001	**< 0.001**
	Audiovisual	Visual only	78.5	0.775	1
	Control	Visual only	484	< 0.001	**< 0.001**
**I was conscious of my real environment**	Friedman test *(df* = *3)*		22.1	< 0.001	–
	Auditory only	Audiovisual	88.5	0.208	1
	Auditory only	Control	8	< 0.001	**0.004**
	Auditory only	Visual	61	0.032	0.193
	Audiovisual	Control	28	< 0.001	**0.002**
	Audiovisual	Visual only	57	0.119	0.714
	Control	Visual only	202	0.011	0.067

During the audiovisual condition, of the 42 participants one reported feeling “completely” unwell, one reported feeling “somewhat” unwell, three reported feeling “slightly” unwell, and thirty-seven reported feeling “not at all” unwell. During the auditory only condition, two participants reported feeling “completely” unwell, one reported feeling “slightly” unwell, and thirty-nine participants reported feeling “not at all” unwell. During the visual only condition, one participant reported feeling “moderately” unwell, one reported feeling “somewhat” unwell, four reported feeling “slightly” unwell, and thirty-six reported feeling “not at all” unwell.

## Discussion

In this study we show that when examining the effects across all three physiological parameters, the results indicate that the audiovisual condition results in the greatest decrease over time. Moreover, participants subjectively reported their preferred intervention to be the audiovisual condition and reported being less aware of their real-world surroundings during this condition, compared to the control condition, or independent inputs.

Regarding the heart rate, participants had the lowest heart rate over the duration of the stimulation when receiving audiovisual stimuli or independent visual stimuli. Moreover, when wearing the head-mounted display, compared to not, participants were significantly less aware of people and light in the study room during the intervention. As such, it is possible that during our study, when the participants received only the head-mounted display, they were able to engage more in the calm virtual setting, compared to when they received only the noise-cancelling headphones^29–31^. This is supported by the notion that vision is more important than hearing for guiding our daily life and can often be enough to effectively substitute real sensory data as it is perceptually dominant^19,29,50^. However, there is evidence to suggest that auditory input may activate areas of the occipital cortex suggesting an important interaction between the auditory and visual cortices during audiovisual stimulation^51^. Moreover, with the known ability of blind individuals to perform better during spatial hearing tasks^52^, future studies may investigate if integrating more advanced auditory stimulus processing into VR stimuli could enhance the VR immersion of users and the relaxing effect presented here.

Looking at the respiration rate, there was an initial drop when the participants first began the intervention. It should be noted that this initial drop, not seen in the heart rate or blood pressure data, is predictable due to the breath-to-breath influence of respiration on autonomic activity^53^. Subsequently, it can be expected based on the respiratory-modulated activity of the heart rate and blood pressure patterns that a more gradual adaptation over time will occur compared to the respiration rate^53^. The faster adaptation of the respiration rate allows differences in the initial changes of the four conditions to be observed. The initial change of the respiration rate during the audiovisual condition is of interest because it shows that when the user is completely removed from the real world around them, they show a more gradual but constant change. During immersive VR individuals must first become accustomed to their virtual surroundings, initially creating a reaction counteracting the expected decrease in respiration rate, before the participants are able to relax into the virtual experience^54^. Accordingly, the subjective data shows that participants reported being more aware of noise and light when wearing only the head-mounted display or the headphones, respectively, compared to when they were receiving audiovisual stimulation. Here it is hypothesized that a lower level of presence was achieved, due to the setup not promoting full immersion.

The finding that the audiovisual intervention provides the greatest mode for relaxation is further supported by the blood pressure which showed that the audiovisual intervention had a significantly stronger relaxation effect than the remaining three conditions. This is in line with the subjective preference of the participants.

Overall, the results of this study suggest that, regardless of age or sex, providing audiovisual inputs to individuals results in a greater objective and subjective relaxation compared to independent inputs or no artificial inputs, which may arise for two main reasons. First, it may arise as a result of the visual and auditory cues being presented simultaneously, allowing the participant to feel more immersed in the virtual nature environment, enabling consciousness of the real-world to be transferred to the virtual world^29^. This transfer of consciousness to the virtual world, also known as presence, would be stronger in the audiovisual condition versus the independent conditions due to the alignment of the stimuli^30,31,33^. This may in turn help to induce the relaxation response known to be elicited via exposure to nature^12–17^. Second, another reason that the audiovisual condition may have resulted in greater relaxation is due to the artificial removal of the real-world surroundings. The audiovisual condition allowed the participants to become subjectively less aware of people, noise, light, and their real-world surroundings, while also providing them with something that they reported enjoying. In this way the audiovisual condition is simultaneously able to tackle the problem of sensory overload and sensory deprivation^35,36^.

## Limitations and outlook

Despite eliciting the relaxation response, a limitation of this study is that it is not possible to determine why this change in physiological values occurs. Whether the changes we observed during the audiovisual intervention occurred because of the proper alignment of the sensory inputs which allowed for a realistic exposure to nature, or due to the devices (i.e., head-mounted display, noise-cancelling headphones) blocking the stressful environment around them is difficult to say. A second limitation of the study is the lack of a proper baseline measurement where the participants were present in the bed for 30 min prior to recording. This is related to another limitation experienced in this study wherein the participants reached a floor effect for relaxation. Therefore, the potential for use of the different stimulation conditions could not be seen as the participants’ resting state was achieved. It would be interesting to determine, for future research, how participants can undergo a full baseline measurement, while maintaining an elevated level of stress. Additionally, it would be of interest to examine if the relaxation effect of immersive VR is able to counter the known negative effects of environments with sensory overload and sensory deprivation, such as in hospitals or confined spaces. This would help to determine the feasibility of using immersive VR as an in-house relaxation tool to counteract stress. Finally, despite detecting significant changes in the physiological parameters that this study examined, the clinical relevance of this has yet to be determined and would be interesting to look into in the future.

## Conclusion

In conclusion, this study has found that based on physiological recordings, participants were objectively more relaxed after receiving audiovisual visual and auditory input, compared to independent auditory or visual inputs, or no artificial sensory input at all. Participants also reported a subjective preference for the audiovisual intervention. Moreover, ratings of awareness also indicated that when receiving the audiovisual intervention, the participants reported being less aware of their surroundings than when receiving only a single input. These findings highlight the importance of the continued use of the audiovisual VR stimulation when aiming to induce an objective relaxation response.

## Supplementary Information

Supplementary Information.

## Data Availability

Study data and code that support the findings of this study are available from the corresponding author, TN, upon reasonable request.
