# Supplementary material for: Investigating the role of auditory and visual sensory inputs for inducing relaxation during virtual reality stimulation
Source: Sci Rep. 2022 Oct 12;12:17073. doi: 10.1038/s41598-022-21575-9 (PMC9560033; doi:10.1038/s41598-022-21575-9)
Supplement: Supplementary file 1 — Supplementary Information. [file 41598_2022_21575_MOESM1_ESM.pdf]

Investigating the Role of Auditory and Visual Sensory Inputs for Inducing Relaxation During Virtual Reality Stimulation

Aileen C. Naef [1,#], Marie-Madlen Jeitziner [2,3,#], Samuel E.J. Knobel [1], Matthias T. Exl[2], René M. Müri[4], Stephan M. Jakob [2], Tobias Nef [1,4,*], Stephan M. Gerber [1]

[1] Gerontechnology and Rehabilitation Group, ARTORG Centre for Biomedical Engineering Research, University of Bern, Bern, Switzerland

[2] Department of Intensive Care Medicine, Inselspital, Bern University Hospital, University of Bern, Switzerland

[3] Institute of Nursing Science, Department of Public Health, Faculty of Medicine, University of Basel, Switzerland

[4] Department of Neurology, Inselspital, Bern University Hospital, University of Bern, Switzerland

#) equal contribution (shared first authorship)

[*] E-mail: tobias.nef@unibe.ch

Supplementary Results

Immersion and presence in the virtual environment

To assess immersion and presence in the virtual environment, during the audiovisual condition, auditory only, and visual only, conditions, questions taken from different validated questionnaires were used (supplementary table S1). The findings of these questions were examined via repeated measures ANOVA with paired t-tests used for the post-Hoc analyses (supplementary table S1). The results supported that there are higher levels of immersion in the audiovisual condition versus auditory only and visual only conditions. They also showed that the audiovisual condition resulted in greater presence than in the auditory only condition. No significant difference was found between the audiovisual condition versus the visual only condition. Based on these results we interpret the findings to suggest that the head mounted display is more important for presence than the auditory component, but both play a role for immersion.

Supplementary Table S1. Results of the presence and immersion questions. The calculated F-statistic and associated p-value (repeated measure ANOVA) for each question, as well as post-hoc analyses (t-test) between conditions.

Question	ANOVA	Post Hoc Conditions	Post Hoc Results
Presence			
**How aware were you of the events occurring in the real world around you?** *Presence Questionnaire Item Stems[45]: Item 8*	F(2.24,99.4) = 8.863 p<**.001**	AV-A	t(41)= -1.16, p.adj= 1
		A-V	t(41)= -2.23, p.adj= 0.187
		AV-V	t(41)= -1.70, p.adj=0.579
		Control-AV	t(41)= -4.27, p.adj< **.001**
		Control-V	t(41)= 2.79, p.adj= **0.047**
		Control-A	t(41)= -4.16, p.adj< **.001**
**In the computer-generated world I had the sense of being there.** *IPQ[46]: Item 1*	F(2,82) =3.87 p= **0.025**	AV-A	t(41)= -2.52, p.adj= **0.046**
		A-V	t(41)= -1.43, p.adj= 0.48
		AV-V	t(41)= 1.52, p.adj= 0.405
		Control – AV	-
		Control-V	-
		Control-A	-
Immersion			
**I become so involved in the virtual environment, that I was not aware of things happening around me.** *User Experience Questionnaire [47]: Item 17*	F(2,82) =6.707 p= **0.002**	AV-A	t(41)= -3.70, p.adj<**.001**
		A-V	t(41)= -1.91, p.adj= 0.188
		AV-V	t(41)= 1.73, p.adj= 0.276
		Control – AV	-
		Control-V	-
		Control-A	-
**I become so involved in the virtual environment that I lose al track of time.** *User Experience Questionnaire [47]: Item 22*	F(2,82) =4.269 p= **0.017**	AV-A	t(41)= -2.78, p.adj=**0.008**
		A-V	t(41)= 0, p.adj= 1
		AV-V	t(41)= 2.73, p.adj= **0.009**
		Control-AV	-
		Control-V	-
		Control-A	-

Effect of time and the effect of the interaction between time and condition

In addition to the main effect of condition on the physiological parameters (presented in the main results), the effect of time and the effect of the interaction between time and condition were also investigated using the generalized additive mixed model (GAMM). These secondary effects were examined based on the results of a maximum likelihood ratio test which showed strong evidence that there are differences in the smooth functions of the model.

The results of the GAMM presented in Table S2 show both the effect of time and the effect of the interaction between the condition and time for the duration of the experiment. Significant results here suggest that the parameter of interest did not remain stable in its change over the duration of the experiment. This can be seen visually in supplementary Fig. S1-S3.

Supplementary Table S2. Results of the effect of time and the effect of the interaction between the intervention condition and the duration of the experiment as predicted by the generalized additive mixed model (GAMM). For each model, the interaction of each intervention condition over the duration of the experiment is examined and presented here. GAMM models do not use linear coefficients, but use smooth functions which are referred to as s(). Abbreviations are as follows: effective degrees of freedom (edf), f-statistic (F), p-value (p).

	**edf**	**F**	**p**
Heart Rate			
***s*(Timepoint)**	8.612	139.985	**< .001**
***s*(Timepoint) * Audiovisual**	0.391	0.070	0.190
***s*(Timepoint) * Auditory Only**	4.752	2.856	**< .001**
***s*(Timepoint) * Visual Only**	0.034	0.004	0.189
***s*(Timepoint) * Control**	4.811	3.486	**< .001**
Respiration Rate			
***s* (Timepoint)**	8.639	25.964	**< .001**
***s* (Timepoint) * Audiovisual**	1.154	4.112	**< .001**
***s* (Timepoint) * Auditory Only**	0.735	0.286	0.054
***s* (Timepoint) * Visual Only**	1.61e-04	4.92e-06	0.359
***s* (Timepoint) * Control**	0.899	0.682	**0.006**
Blood Pressure			
***s* (Timepoint)**	4.62	21.792	**< .001**
***s* (Timepoint) * Audiovisual**	1.97e-05	2.30e-08	0.940
***s* (Timepoint) * Auditory Only**	7.78e-05	1.68e-06	0.659
***s* (Timepoint) * Visual Only**	0.320	0.052	0.227
***s* (Timepoint) * Control**	6.74e-05	7.36e-07	0.757

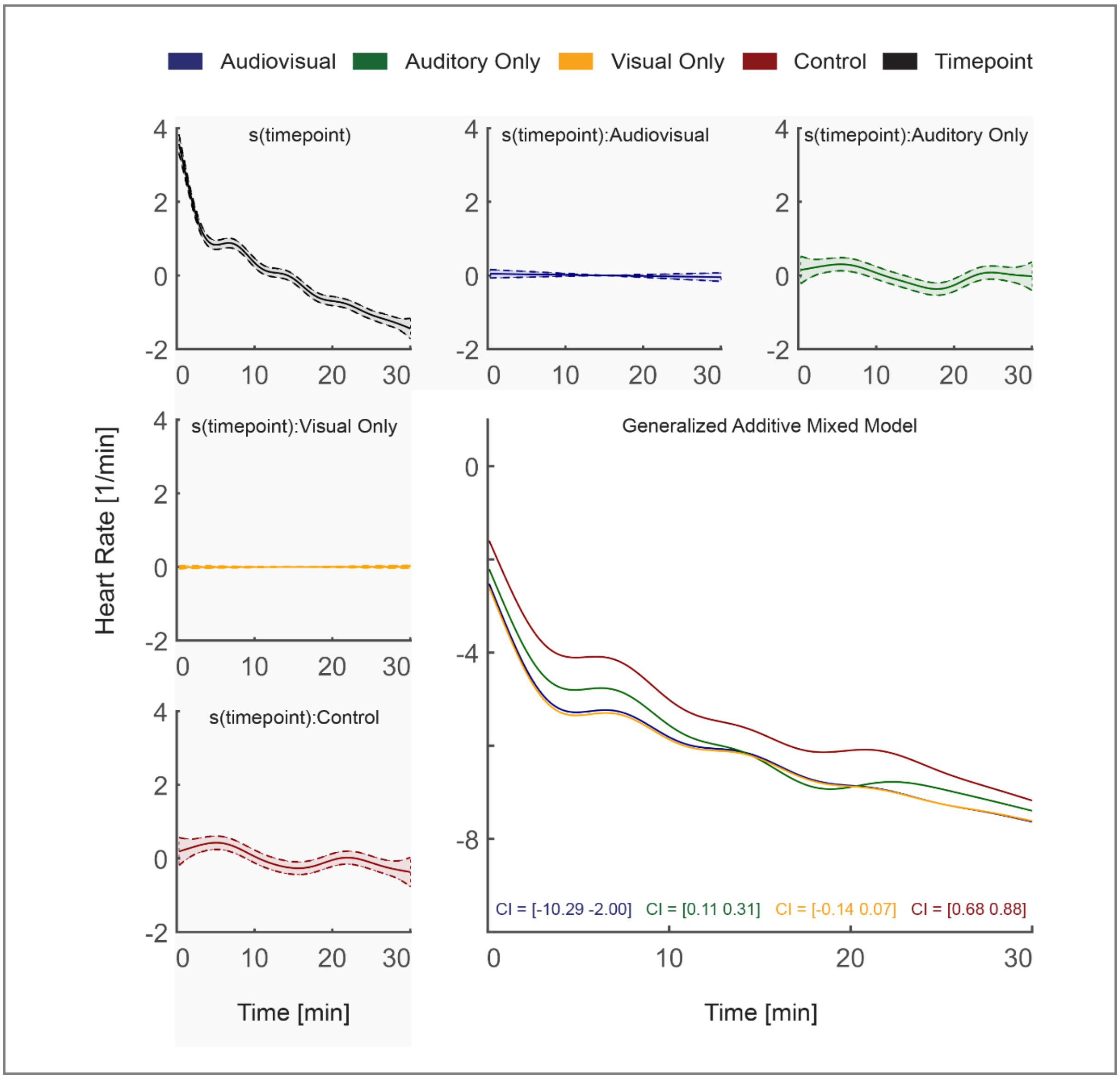

Supplementary Figure S1. Effect of time and effect of the interaction between condition and the duration of the experiment on the heart rate (1/min). The five smaller plots shown in the shaded area represent the smoothing terms considered by the generalized additive mixed model (GAMM). Dashed lines and shaded areas represent the 95% confidence intervals. The larger plot represents the predicted values of the heart rate, generated by the model. The y-axes of all plots represent the change in heart rate [1/min], while the x-axes of all plots represent the time [min]. (Top Left) Effect of time, smooth function for time – shows that not taking into consideration the different conditions provided to the participants, heart rate decreases over time. (Top Middle) Effect of interaction between the audiovisual condition and the duration of the experiment. No significant effect was found, which is consistent with the horizontal line. This suggests that the change in heart rate elicited by the audiovisual condition remained constant over time. (Top Right) Effect of the interaction between the auditory only condition and the duration of the experiment. There was a significant effect found which shows that during the auditory only condition the heart rate fluctuated (i.e., increased and decreased). (Middle Left) Effect of interaction between the visual only condition and the duration of the experiment. No significant effect was found, which is consistent with the horizontal line. This suggests that the change in heart rate elicited by the visual only condition remained constant over time. (Bottom Left) Effect of the interaction between the control condition and the duration of the experiment. There was a significant effect found which shows that during the control condition the heart rate fluctuated (i.e., increased and decreased). (Bottom Right) Modelled data generated using the GAMM showing the effect of condition on heart rate. Numbers in the bottom of the plot show the confidence intervals (CI) for each condition.

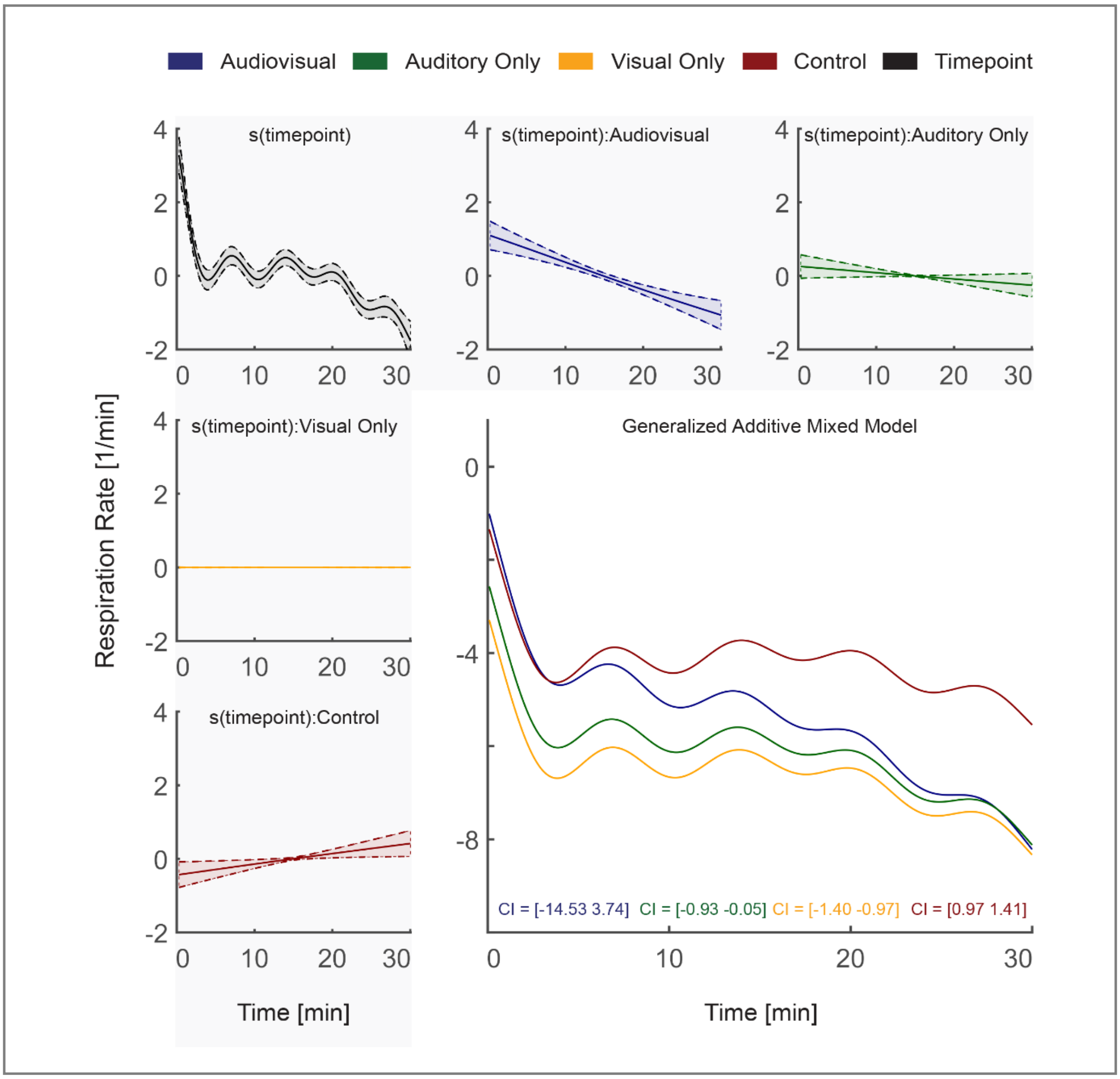

Supplementary Figure S2. Effect of time and effect of the interaction between condition and the duration of the experiment on the respiration rate (1/min). The five smaller plots shown in the shaded area represent the smoothing terms considered by the generalized additive mixed model (GAMM). Dashed lines and shaded areas represent the 95% confidence intervals. The larger plot represents the predicted values of the respiration rate, generated by the model. The y-axes of all plots represent the change in respiration rate [1/min], while the x-axes of all plots represent the time [min]. (Top Left) Effect of time, smooth function for time – shows that not taking into consideration the different conditions provided to the participants, respiration rate decreases over time. (Top Middle) Effect of interaction between the audiovisual condition and the duration of the experiment. There was a significant effect found which shows that during the audiovisual condition the change in respiration rate did not remain constant. (Top Right) Effect of the interaction between the auditory only condition and the duration of the experiment. No significant effect was found, which is consistent with the relatively horizontal line. This suggests that the change in respiration rate elicited by the auditory only condition remained constant over time. (Middle Left) Effect of interaction between the visual only condition and the duration of the experiment. No significant effect was found, which is consistent with the horizontal line. This suggests that the change in respiration rate elicited by the visual only condition remained constant over time. (Bottom Left) Effect of the interaction between the control condition and the duration of the experiment. There was a significant effect found which shows that during the control condition the respiration rate did not remain constant. (Bottom Right) Modelled data generated using the GAMM showing the effect of condition on respiration rate. Numbers in the bottom of the plot show the confidence intervals (CI) for each condition.

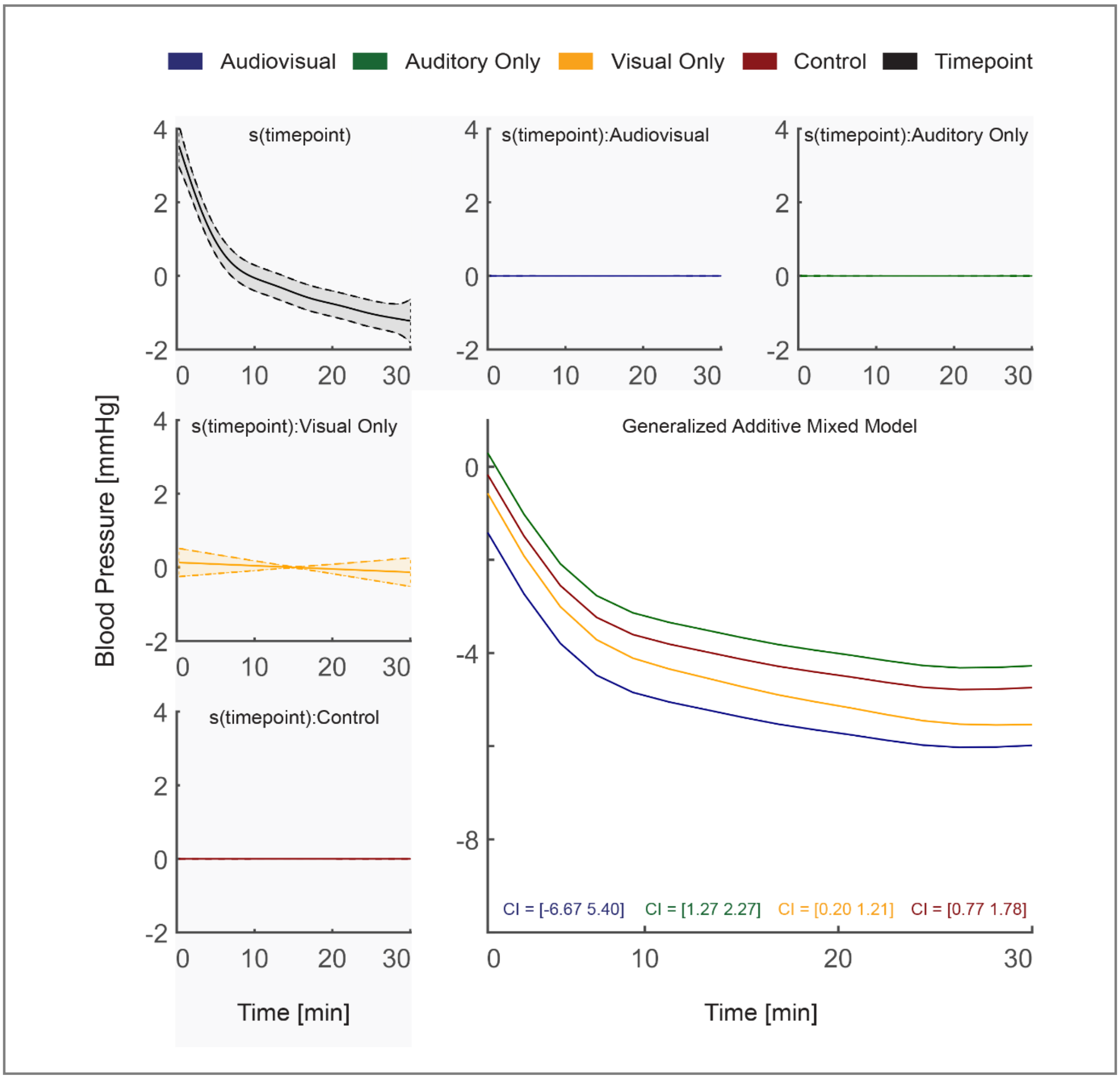

Supplementary Figure S3. Effect of time and effect of the interaction between condition and the duration of the experiment on the blood pressure (mmHg). The five smaller plots shown in the shaded area represent the smoothing terms considered by the generalized additive mixed model (GAMM). Dashed lines and shaded areas represent the 95% confidence intervals. The larger plot represents the predicted values of the blood pressure, generated by the model. The y-axes of all plots represent the change in blood pressure [mmHg], while the x-axes of all plots represent the time [min]. (Top Left) Effect of time, smooth function for time – shows that not taking into consideration the different conditions provided to the participants, blood pressure decreases over time. (Top Middle) Effect of interaction between the audiovisual condition and the duration of the experiment. No significant effect was found, which is consistent with the relatively horizontal line. This suggests that the change in blood pressure elicited by the audiovisual condition remained constant over time. (Top Right) Effect of the interaction between the auditory only condition and the duration of the experiment. No significant effect was found, which is consistent with the relatively horizontal line. This suggests that the change in blood pressure elicited by the auditory only condition remained constant over time. (Middle Left) Effect of interaction between the visual only condition and the duration of the experiment. No significant effect was found, which is consistent with the horizontal line. This suggests that the change in blood pressure elicited by the visual only condition remained constant over time. (Bottom Left) Effect of the interaction between the control condition and the duration of the experiment. No significant effect was found, which is consistent with the horizontal line. This suggests that the change in blood pressure elicited by the control condition remained constant over time. (Bottom Right) Modelled data generated using the GAMM showing the effect of condition on blood pressure. Numbers in the bottom of the plot show the confidence intervals (CI) for each condition.
